# The Hospital. Nursing Section

**Published:** 1905-05-20

**Authors:** 


### The Hospital.

### Nursing Section.

Contributions for this Section of "The Hospital" should be addressed to the Editor, "The Hospital" Nursing Section, 28 & 29 Southampton Street, Strand, London, W.C.

No. 973.—Vol. XXXVIII. SATURDAY, MAY 20, 1905.

### Notes on News from the Nursing World.

### THE ARMY SERVICE RESERVE.

A statement has been made that there is a new reserve section of the Army Nursing Service. This is not the case. The old Army Nursing Service Reserve still exists, and the only alteration which has been made is in respect to the pay. This has been increased in order to bring it up to the new rates of pay drawn by the members of Queen Alexandra's Imperial Military Nursing Service. Of course, the members of the Reserve have to carry out the new regulations of the regular service when they are called up for duty and to accept the additional responsibilities involved, such as managing their own wards, superintending the cleanliness of them, and looking after the equipment. In return for this they get gratuities, varying from £15 to a matron, £10 to a sister, and £7 10s. to a staff nurse, as well as excellent pay. Princess Christian remains the president of the Reserve, but when the members are required to be on duty they come under the jurisdiction of the War Office, and the control of the nursing board of which the Queen is president.

### RESIGNATION OF A LONDON MATRON.

We are informed that Miss Zoe Longstaff, matron of Mount Vernon Consumption Hospital, Hampstead, has tendered her resignation. The connection of Miss Longstaff dates back six years. For three of these she was sister, and in 1902 she succeeded Miss Whitton as matron. During her term of office she has reorganised and amplified the course of training for probationers at the hospital, so that they now enjoy the advantage of a systematic course of lectures and classes, beside instruction in theoretical and practical pharmacy. Her retirement is not due to ill-health, but to circumstances of a personal character.

### THE SITUATION IN SOUTH AFRICA.

It appears that nurses are still going out to South Africa to swell the ranks of the insufficiently employed. Since about December last there has been the usual improvement which always takes place at this time of the year in nursing prospects, due in the main to the sick season in Cape Colony, which is now passing over. In the Transvaal there has not been even a temporary change for the better. We hear on good authority that there are 800 nurses in Johannesburg and work for 200, and that nurses travel from Johannesburg to Cape Town in the vain hope of getting work. Yet fresh nurses continue to arrive from home, sometimes with introductions to doctors, thus obtaining, probably only for a short time, patients at the expense of others equally well qualified and already acclimatised. Last year many nurses in the Cape Colony were unable for quite six months to earn more than a living wage, and others had to draw upon their savings. Of two who went out from England to join a recognised institution, one in her first six months had three months' work. The other, with the highest recommendations, was barely able to make her way, £1 13s. 6d. a week being the cheapest rate for board and bedroom accommodation, shared with one or more others, and 7½ per cent. being taken off fees of £3 3s. 0d. a week for institution charges. It is easy to understand that even those who are regularly employed in South Africa are not on the high road to fortune.

### THE SIGNING OF DOCUMENTS.

A correspondent who asks whether, while the question of State registration is on the tapis, something could not be done to make the co-operative system more general, in order to render nurses less dependent upon mere money-making institutions, states that in many cases applicants for employment are required to sign a document which threatens them with a penalty of £50 should they attempt to work for their own fees within a radius of, sometimes, 20 miles. She adds that "of course the document is not binding, but many nurses are not aware of this." There is no "of course" in the matter. The proviso as to the penalty may be oppressive and unjustifiable, but an agreement is an agreement whatever the nature of its conditions, and a person who violates it is liable to any penalty prescribed. The remedy is for no fully-trained nurse to consent to put her name to a document which, on the face of it, is unreasonable.

### PLYMOUTH GUARDIANS AND THEIR CHARGE NURSE.

A new question has arisen at Plymouth. A charge nurse at the infirmary has resigned, and though the Guardians at their meeting decided that her letter should not be read, they accepted her resignation. In this communication she complains that she has experienced great difficulty in retaining her position owing, as she alleges, to rudeness on the part of assistant nurses and to the failure of the Hospital Committee to uphold her. She states that the superintendent nurse and the medical officer have always been very helpful and considerate to her, both when she was in the infirmary as assistant nurse for a year and since she has been charge nurse for a year and five months. One of the Guardians, in referring to her letter, said that "as the whole matter of the nursing staff was sure to come before the Local Government Board it would be very unwise to make anything in the way of evidence public," and we think that there is force in his argument. But the resignation of the charge nurse emphasises the need for an inquiry, and the sooner it takes place the better.

### REGISTRATION IN THE DRAWING-ROOM.

There was a drawing-room meeting at Londonderry House on Tuesday, presided over by the Marchioness of Londonderry, at which speeches in favour of the State Registration of nurses were delivered by the matrons of St. Bartholomew's Hospital, the Royal South Harts Hospital, and other ladies whose views are well known. A resolution in favour of forming a lay association for promoting registration was carried and several names were approved as members. The proceedings were not entirely unanimous, one lady urging the advisability of having at least two grades of registration, and another contending that more classification was desirable.

### SPECIAL TRAINING SCHOOL FOR INFECTIOUS DISEASES.

The importance of nurses acquiring a knowledge of the nursing of infectious diseases has been recognised in Victoria in a satisfactory manner. Until recently none of the nurses in the colony but those trained at the Melbourne Hospital could claim to have had experience in the nursing of infectious diseases. But there has just been established at Queen Victoria Memorial Hospital for Infectious Diseases at Fairfield a course of four months' duration, open to all nurses on the general register of the Royal Victoria Trained Nurses' Association. It embraces practical work in the wards, instruction and lectures from the matron, and courses of lectures by the medical staff. Courses on a similar basis might with advantage be arranged at some of the hospitals for infectious diseases in England, open only to nurses who have obtained their certificates of general training for three years.

### AN ESSEX GUARDIAN AND TRAINED NURSES.

At a recent meeting of the Chelmsford Guardians it was decided that for the present the six nurses employed at the Infirmary were sufficient, and that it was not necessary to advertise for a probationer. The Medical Officer, however, stated that if the Board did not authorise a nurse to be advertised for he would have to order one in the next day, and he had accordingly engaged one from the Nurses' Home at Chelmsford at 30s. a week. The Guardians maintained at a subsequent meeting that in so doing he had acted in defiance of them, but in defending himself the Medical Officer said that he had simply made use of the power which he possessed in order to provide sufficient nurses until the next Board meeting, the nurse having been engaged only till that day. The Assistant-Clerk reminded the Guardians that in a case of emergency the doctor was allowed to get extra nursing help, and that it was entirely for him to decide whether the emergency existed. After an animated discussion, in which it was shown that the Local Government Board Inspector had not considered that the Infirmary was overstaffed with seven nurses, but had expressed himself well satisfied with the arrangements, observing that the Infirmary was "one of the best regulated in the Eastern Counties, and that they had an excellent Superintendent Nurse," it was decided to advertise for another probationer. This was decided upon notwithstanding the statement of one of the Guardians that, speaking from illnesses that he had seen, he considered that people did not want to be bothered with professional nurses, who "came and turned you over one way then the other, then washed you, then pulled you out of bed, etc., so that it was almost impossible for any one to recover." It is to be hoped that there may still be a "Gamp" to be found for this gentleman should he unfortunately one day contract an illness. No trained nurse would like to feel that by her presence his chances of pulling through had been seriously jeopardised!

### TIDING OVER A CRISIS.

It was stated at the annual meeting of the Derbyshire County Nursing Association that the organisation had just passed through a very critical period. At the end of 1903 it was so poorly off financially that the managers had to draw upon the small reserve fund of £100. In the following year the donations were so considerable that it became possible to replace the reserve fund, and start afresh with a credit balance. But we agree with the treasurer that the association cannot expect to exist simply by means of donations; and as the number of subscribers is only sixty, it certainly ought to be materially increased. During the past year two new centres were formed, one at Spondon and the other at Stanton-by-Dale and Dale Abbey. Arrangements are being made for other extensions, and the association is fortunate in having for its nurse superintendent Miss S. A. Thorpe, who has lately been appointed inspector of midwives under the Derbyshire County Council.

### QUEEN ALEXANDRA'S MILITARY NURSING SERVICE.

We are officially informed that Miss E. C. Macpherson and Miss A. Ayre have been appointed staff nurses in Queen Alexandra's Imperial Military Nursing Service. The following staff nurses have been promoted to be sisters:—Misses A. R. F. Auchmuty, S. K. Bills, B. N. Daker, G. Knowles, C. Mackay, W. G. Massey, B. Rennie, and M. Worthington. Miss E. M. Keays has resigned her appointment as staff nurse. Staff Nurses H. Hartigan, H. M. E. Macartney, and E. M. Rentzsch have been posted to the Royal Herbert Hospital, Woolwich; Staff Nurse M. J. Hepple to the Cambridge Hospital, Aldershot; and Staff Nurse K. Roscoe to the Royal Victoria Hospital, Netley. Miss Beatrice Jones, matron, has been transferred to the Military Hospital, Millbank, S.W., from the Royal Herbert Hospital, Woolwich. Sister A. Barker has been transferrred to Chatham from Station Hospital, Rochester Row; Sister L. M. Culverwell to the Military Hospital, Millbank, from the Station Hospital, Rochester Row; Sister C. G. Stronach to Egypt from Connaught Hospital, Aldershot; Sister A. R. F. Auchmuty to the Connaught Hospital, Aldershot, from the Cambridge Hospital, Aldershot; Sister C. Mackay to the Military Hospital, Millbank, from the Royal Herbert Hospital, Woolwich, and Sister E. M. E. Todd, to the Connaught Hospital, Aldershot, on her return from South Africa. Staff Nurse S. O. Beamish has been transferred to the Royal Herbert Hospital, Woolwich, from Shorncliffe; and Staff Nurses L. Cunningham, A. M. Orchard, and E. M. Robinson to the Military Hospital, Millbank, from the Royal Herbert Hospital, Woolwich. Staff Nurses G. M. Allen, K. M. Bulman, N. Blew, L. M. Dann, E. Foster, M. E. M. Grierson, A. M. Orchard, M. S. Ram, and M. F. Steele have been confirmed in the appointments.

### ROYAL VISIT TO ST. MARY'S HOSPITAL.

On Tuesday morning the Princess Beatrice of Coburg, with Miss Minna Cochrane in attendance, visited St. Mary's Hospital, Paddington. The Princess was welcomed by the chairman, Mr. H. A. Harben, L.C.C., the senior surgeon, Mr. Herbert W. Page, the matron, and the secretary, all of whom were presented by the Rev. H. Russell Wakefield to her. Her Royal Highness made a thorough inspection of the hospital, went through the old building, which is just now crowded with patients, the new Clarence Memorial wing, and the new out-patients' department. The Princess appeared very pleased with what she saw, especially with the ward offices in the new wing, which are a great improvement on the old-fashioned arrangements in the old hospital. Her Royal Highness also inspected with much interest one floor of the new home for nurses, situated at the top of the building, which will be opened shortly, with its spacious sitting and class-rooms and its comfortable single bedrooms, one for every nurse.

### RE-UNION AT CHELSEA HOSPITAL FOR WOMEN.

On Wednesday evening last week, Chelsea Hospital for Women was the scene of a happy meeting of sisters and nurses past and present. A musical evening was got up under the kind and able help of Miss Florence Christie, who also brought with her Miss Sasse, a brilliant pianist, and Miss Hesse, a pleasing reciter. The nurses, conducted by Miss Florence Christie, sang several glees with spirit and a good deal of talent. At the close of the concert, after the departure of the guests, Dr. Victor Bonney was presented with a mahogany bureau as a wedding gift by the matron and nursing staff.

### A NURSES' LETTER CLUB.

It may interest some of our readers to learn that a Circular Letter Club has been for some time in existence in Canada. Originated in 1901 by the lady superintendent of Mack Training School and General and Marine Hospital, St. Catherine's, Ontario, it appears to have obtained a certain measure of popularity in the Dominion. It was started by making a numbered list of the names and addresses of those nurses who were to form the club, the names being written and numbered in the order that was deemed most desirable for the letter to travel. No. 1 having written the first letter wrote to the others who were to comprise the circle, and sent the letter and the list of names and addresses to No. 2 on the list. No. 2 opened and read, wrote a letter and put her letter along with the one she received and the list into a new envelope and sent to No. 3, and so on from one to another until it reached the last on the list in the club, No. 16. After writing her letter No. 16 sent the whole packet to No. 1, thus completing the circle. This club letter travelled upwards of 20,000 miles, during a period of several months, and beginning with Orillia in Ontario went westward across the American Continent and the wide Pacific to the Philippine Islands, including in its tour the Rockies, British Columbia, Port Huron, Detroit, Iowa, and Tennessee. When it was eventually received by No. 1, she took out of the budget her own epistle, which had been read and commented upon by each one of her friends, and after writing a new letter, started the thus fully-fledged circular letter on a second round. The 16 nurses have steadily maintained it, and consider it an excellent scheme for keeping a number of friends living in different places, widely scattered, in touch with one another.

### NURSES' STALL AT THE WESTMINSTER HOSPITAL BAZAAR.

One of the most attractive features of the great bazaar to be held in Dean's Yard on Tuesday, Wednesday, and Thursday next week, in aid of Westminster Hospital, will certainly be a flower-stall presided over by the matron. Miss Cave will have the assistance of the sisters and nurses of the hospital, who will take their turn during off-duty time in promoting the sale of button-holes and bouquets among the visitors, who are likely to be exceedingly numerous.

### HOSPITAL FOR MOTHERS AND BABIES.

Under very auspicious circumstances the Hospital for Mothers and Babies, which is also intended to promote the training of gentlewomen as district midwives, was opened last week at Woolwich. The opening ceremony was performed by Princess Christian, who received an address from the Committee explaining the aims and scope of the proposed work. Dr. Cullingworth was able as a member of the Central Midwives Board to commend the undertaking, and he expressed his sympathy in warm terms, which shows that he fully endorses the statement that it has been rendered necessary by the passing of the Act of 1902. Full details are given in another column. Miss Alice Gregory, the honorary secretary, who has worked so hard in the cause, must be congratulated upon seeing the beginning of the enterprise which she originated carried into effect. We do not doubt that if it fulfils the expectations which have been generally formed respecting it, and tends to bring about the employment of a superior class of women in midwifery, the financial support essential for the completion of the project will be forthcoming.

### SHORT ITEMS.

Miss Marion Dashwood, hon. secretary of the Nurses' Union, is giving a series of Bible Readings at 26 George Street, Hanover Square, on Tuesday evenings during the months of May and June from 6 to 7 p.m., to which all nurses are cordially invited.—The third annual meeting of the Nurses' Missionary Union will be held at University Hall, Gordon Square, on Thursday next, from 3 to 9 p.m. in three sections. Miss Kathleen Miller will read a paper at the evening meeting on "A Nurse's Opportunities."

### The Nursing Outlook.

"From magnanimity, all fear above;
From nobler recompense, above applause,
Which owes to man's short outlook all its charm."

### COMBINED DISTRICT NURSING AND MIDWIFERY.

The combination of the work of midwife with that of district nurse may doubtless appear to be convenient in small urban and rural districts. It is, however, open to serious objection, and the arrangement would be fraught with dangers that ought if possible to be avoided. These dangers centre chiefly round the question of conveying infection and the additional risks to which lying-in women may be exposed when they are attended in their confinements by women who work not only as midwives but also as district nurses.

With a view to the prevention of puerperal fever, the Central Midwives Board have recently issued printed directions to midwives. In the first paragraph of these directions the midwife is told that "the smallest particle of decomposing matter may set up puerperal fever." This fact needs to be strongly emphasised. It is not yet sufficiently well known to midwives and to the general public that contact with all forms of decomposing organic matter is inimical to health. During the process of decomposition and the dissolution of organic substances destructive micro-organisms of various kinds are produced in large numbers. When the resisting force of the cells of the body is feeble, as it is in the case with young children, or when it has been temporarily reduced in a woman by the strain incidental to childbirth, a small degree of virulence possessed by these destructive agents may be sufficient to overcome the resisting or constructive force of the cells; and, in the case of a lying-in woman, puerperal fever may then be produced.

Under existing conditions it may not always be possible for the midwife to entirely avoid all contact with septic or decomposing matter. Nevertheless, that is the goal towards which she must strive, and which she needs constantly to keep in mind, if puerperal fever and other septic troubles are to be prevented. With respect to district nursing, it is obvious from the nature of the work that the district nurse is brought more frequently and more constantly into contact with various kinds of decomposing organic matter than the woman who acts only as midwife. One great objection, then, to allowing a woman to combine midwifery and district nursing is this necessity for contact with decomposing matter to which a district nurse is liable. And the peril is the greater the less thorough the hospital training and experience of the district nurse have been. If she be a fully-qualified, conscientious, district nurse, with two or three years' hospital training and experience (a Queen's nurse, for example), her knowledge and experience in the use of antiseptics, and in surgical cleanliness, will greatly lessen the risk of her carrying infection. But the risk cannot be altogether avoided by attention to cleanliness and the use of antiseptics, especially in those cases where the district nurse is brought into daily contact with septic matter of a virulent type; such as the offensive discharges of cancer of the uterus or of the breast, of bad chronic ulcers of the leg, or of bad bedsores due to disease or to neglect in old bedridden people; the pus which flows from an abscess of the breast, or from abscesses and sinuses caused by tubercular hip-disease or by bone disease.

The combination also constitutes a danger because it sets a bad example to the untrained ignorant *bonâ fide* midwife. She is too ignorant, and sometimes too self-satisfied, to draw a line of distinction between herself and the trained district nurse, or to understand the value of surgical cleanliness and the use of antiseptics. If the district nurse may tend the sick and the dying and go to confinements, why may not she too? Such will be her argument, and it will satisfy her conscience, for she sets small value on antiseptics and on what she calls "these *new* ways."

Yet another difficulty arises out of a custom in connection with the laying out of the dead. When a patient whom a district nurse has been attending dies, it is customary for her to lay out the dead. So long as this custom remains she cannot add to her duties those of a certified midwife without breaking the rule of the Central Midwives Board, which states that "no midwife shall undertake the duty of laying out the dead."

Of course we know that owing to innovations introduced by the Midwives Act, the practice of midwifery by midwives is in a transitional stage to-day, and that during transitional stages unforeseen temporary local difficulties often arise. These tend gradually to die out as the new order of things becomes more firmly established; and it is unwise to set new precedents, during transition periods. District nursing and midwifery cannot be safely combined unless or until provision can be made for adequate, competent supervision of the district nurses, and for a careful selection of surgical and medical cases which the district-nurse-midwife may attend.

### The Nursing of Sick Children.

By James Burnet, M.A., M.B., M.R.C.P.Edin., Registrar, Royal Hospital for Sick Children; Clinical Tutor, Extramural Wards, Royal Infirmary; and Physician to the Marshall Street Dispensary, Edinburgh.

### VII.—ON THE MANAGEMENT OF RHEUMATIC CASES.

Rheumatism, in all of its varied forms, is a very common disease in childhood. It does not always assume the common type met with in the adult. Thus joint affections are much less severe as a rule in the child, while the throat is more liable to be attacked. Excessively high temperatures are occasionally met with in the adult, whereas in children suffering from rheumatism this phenomenon, known as hyperpyrexia, is practically never met with. The nurse who is told that a certain child under her charge is suffering from rheumatism will, therefore, frequently be disappointed if she expects to find much involvement of the joints, or, indeed, a picture of the disease as it is presented to us in later life.

When rheumatism attacks a child it may affect him in many ways. He may have simply slight pains in his limbs, or he may have a sore throat in addition. He may likewise have curious skin eruptions which can only be accounted for by the fact that they occur in a rheumatic subject. Again pleurisy may be a rheumatic manifestation, as may also affections of the heart. In fact the latter are specially liable to occur in childhood. Very frequently we meet with patients who are breathless and bloodless, and yet have no definite history of rheumatism beyond the fact that, some time before, they suffered from a sore throat and slight pains in the lower limbs. The heart is found to be enlarged, and the valves diseased; and we can arrive at one conclusion only, namely, that the child has actually had an attack of rheumatism which has left its permanent effect upon the heart. St. Vitus's dance or chorea, as it is technically termed, is undoubtedly a rheumatic condition in the majority of cases, and in this disease also the heart is very often found to be seriously affected. It must always be remembered, therefore, that the heart is the chief organ which is liable to be attacked in the course of rheumatism as it occurs in children.

Acute rheumatism is a febrile disease—in other words, it will be found that the child's temperature is raised. It is seldom, however, very high, averaging, as a rule, from 102° to 103° F. In view of this fact the child must be kept warm. It is important to see that he wears a flannel nightdress, which should button up the front all the way from top to bottom, so as to admit of ready examination of any part of the child's body without having to move him during the process. He should also sleep between blankets. The reason for this precaution is that sweating is usually profuse, and the patient would consequently become quickly chilled were he to lie between cotton sheets. He should be moved as little as possible, as the slightest movement readily occasions great pain in cases of rheumatism. If the sweating is very profuse, the child should be sponged over night and morning with tepid water, care being taken to dry the skin carefully afterwards. This prevents the development of sweat rashes, which are often a source of annoyance to the patient.

The diet must be carefully attended to. At first only milk and soda-water should be allowed. Later the child may have milk puddings, chicken soup, and some dry toast; but broths and butcher's meat should be withheld entirely until convalescence has become fairly well established. The bowels should be made to move every day, as constipation is apt to lead to straining, which is undoubtedly very bad for the heart. The urine is generally muddy, and deposits either a whitish or pinkish sediment. Should the urine prove irritating and the child complain of pain on passing it, this fact ought to be reported to the physician, and meantime barley water and abundance of lemon juice should be given the patient to drink.

All painful joints must be carefully rolled up in cotton-wool, which may be fastened to the limb by means of a domette bandage. The pressure of the bedclothes is to be avoided as far as possible, as this serves to increase the pain, as does also any attempt to move the limb. In specially severe joint affections it may be necessary to use a splint, to which the limb is loosely bandaged in order to keep it at rest, and so prevent all possible movement.

The pulse must be carefully watched in all rheumatic cases. Special note should be taken of any sudden quickening of the rate or irregularity in the rhythm. These may indicate the commencement of heart involvement, especially if the child appear to have pain in the chest. As a rule, however, heart complications become evident only when the patient becomes restless and appears to have some embarrassment of his breathing. Invasion of the heart is always to be suspected in rheumatic cases when the child cannot breathe freely unless his shoulders and head are raised, and when, in addition, an irritating cough shows itself. In bad cases the lips may become blue and the urine scanty, while the ankles may become puffy and dropsical. The other symptoms, however, of heart affection need not be referred to at greater length in this lecture. It is the nurse's duty in all such cases to immediately report any alteration in the pulse or respiration, or any evidence of pain in the chest to the medical attendant, in order that he may satisfy himself as to the state of the heart and circulation.

In view of the fact that the heart is readily attacked in rheumatic patients, any attempt at sitting up or making sudden movements should be at once prevented by the nurse, who must give special heed to these necessary precautions in every case. In lifting the patient in order to shake up the bed this may be accomplished without causing any discomfort if the child is simply raised in the blanket, which may be made into the form of a hammock for this purpose. In this the patient may be gently raised and placed in another crib or bed until such time as his own has been thoroughly aired and shaken up. The aim of the nurse should be to prevent the patient from putting forth the slightest exertion, as any form of muscular effort is apt to prove a serious strain upon the heart and circulation.

When the attack is over the child will remain pale and bloodless-looking for a considerable time. He should be warmly clad with woollen garments next the skin, long thick stockings and well-soled boots, even in summer weather. Any vigorous exercise is liable to prove dangerous, though short walks may be taken every day during fine dry weather with advantage. If the heart has been affected the child should be most carefully nursed, and strict attention paid to his diet and gastro-intestinal tract for several months after convalescence has become established. Late hours and all school work must be avoided if the heart is to be given a chance of recovering its tone. Such cases are apt to become somewhat chronic, and it is only by the most careful nursing and supervision that they can be tided over what is undoubtedly a critical and grave disease.

We have said nothing regarding medicines, as this part of the treatment belongs, of course, to the physician in attendance. It may be well, however, if we point out that the drugs commonly given for rheumatism are very liable to produce deafness and noises in the ears. The nurse must not be suprised, therefore, if the patient become suddenly deaf during the course of an attack of rheumatic fever, and she should at once make a note of this with a view to subsequently intimating the fact to the physician.

### The Nurses' Clinic.

### SOME METHODS OF TREATMENT FOR PATIENTS' BACKS.—BY A SISTER.

The best way of treating a patient's back is often a matter of controversy amongst nurses. Whether in the first stage of redness and tenderness, or when, unfortunately, the skin has broken, the following methods, the results of which have in nearly every case proved curative may be of interest and use, but it is quite possible that some who read this article may think it unnecessary to harp upon the theme because they may know of some treatment which has proved equally beneficial and good. There will, however, be those who are commencing their training and have as yet only their theoretical knowledge to guide them; to such the experiences of others of some years' training will be welcome.

To secure absolute comfort for the patient who must lie upon his back for a long time, the back must be kept in as good condition as possible, and this will only be done by constant care on the part of the nurse.

To add to his pain that of a sore back not only casts a reflection on the nurse but also often prolongs illness.

Taking a patient who has only been in bed for a short time, the condition of the back is no doubt perfect, and to keep it so attention must be given to it night and morning, first by washing the skin with warm water and plenty of soap, then by drying it and rubbing in gently and firmly some spirit—brandy or methylated spirit will do; these help to harden the skin—powder with starch or a mixture of starch and zinc powder. It is the rubbing which is so necessary; the applications are of little avail without, the circulation is thereby improved, and the stagnation, which in time causes a sloughing of the tissues, becomes impossible. This must be well understood and remembered by the nurse who would ward off the dreaded soreness. If there is constant dampness from incontinence or the nature of the operation or disease, then use some ointment. Ung. zinci is very good; it should be well rubbed in with the hand. If a nurse takes on a case which has either been neglected or unskilfully nursed she will most probably have to deal with a back which has become very sore, the skin broken and a raw wound presenting itself for treatment. First and foremost, pressure must be removed as far as possible; if it be allowed the patient should be placed on his side, propped over with a firm pillow, or, if he may not turn, then the patient must be put on a water-bed. Where this is not practicable an ordinary air-cushion—of the ring kind—may be used with great advantage.

The irritating pressure having been removed the proposed remedies may have a chance. The back should be gently and thoroughly bathed with weak boracic lotion—it will heal much more quickly if kept perfectly clean—then syringed or swabbed with lotio rubra. Next apply on a piece of lint, cut the exact size of the wound, some tinct. benzoin ointment, thinly spread, and over this more lint or wool, to keep the dressing in place; fasten on, either with a T bandage or small strips of strapping—Mead's strapping is very good. Use the method for securing the dressing most suited to the patient. This dressing should be done three times a day in a very bad case, and should be carried on until the wound shows signs of healing. When it is quite dry and healthy looking, then probably powder will be sufficient.

In one case where the back had broken down and the process of healing was very slow, peptone-powder was applied with good result.

In another case a mixture of castor-oil and zinc ointment was used for a back. The patient was thin and badly nourished, and the oil was suggested as a means of feeding up the lax tissues; it certainly answered very well, and has been used on subsequent cases with benefit.

It is always a great source of satisfaction to a good nurse to be able to say at the end of a long and trying illness that her patient has never had the pain of a sore back to add to his discomfort.

This alone will be a good recommendation to any nurse, because it points to care in details and a genuine interest in the welfare of her patient. I do not say that a patient's back never gets sore even if properly attended to, because in spite of all care, sometimes the worst happens.

This, however, is due to the bad condition of the patient probably, and then the best methods should be used in the best possible way.

The worse the case the more triumph for the nurse, and if by using those methods and knowing how to do so, she comes off victorious, then she has knowledge and experience of the greatest practical value.

### Nursing in the United States.

The tendency throughout America seems to be to increase the number of subjects and the term of preliminary probation for candidates who propose to qualify as nurses. At the Massachusetts General Hospital Training School it has been decided to increase the term of probation to six months. The candidates will devote four months of the period to the study of household arts, chemistry, bacteriology, anatomy, and physiology, and the last two months are to be spent in the wards of the hospital Candidates who enter for this preliminary course pay a fee of £10, in addition to £2 deposit for breakages. The deposit is instructive, and indicates that inexperience works out in practice pretty much the same all the world over.

### An Inspector of Training Schools.

On the authority of *The American Journal of Nursing* we stated that the Governor of Maryland had appointed Miss Ross as the Inspector of Training Schools for this State. This announcement was incorrect. The same journal now intimates that Miss Ross was requested by the Board of Nurse Examiners to visit the training schools of the State and look into the methods in vogue, and so to put the Board in a position to judge of the character of the work being done in these schools. Miss Ross's inspection was therefore unofficial in the sense it was originally reported, but she was everywhere cordially received by the training school authorities, and the results are reported to be entirely satisfactory.

### Two Registration Bills Rejected.

On March 25th we reported that the Bill for the Registration of Nurses before the Massachusetts Legislature on the petition of the State Nurses' Association had encountered considerable opposition on the part of the small and special hospitals, particularly for the insane. As originally drawn, this act practically excluded the latter, but amendments were subsequently made which met many of the objections, and liberal concessions satisfied the majority of the opposition. There remained, however, the fundamental objection of the opponents, that the Massachusetts Bill insisted upon the qualification of not less than two years in a hospital. This essential provision, which is supported by the best nursing opinion in this country, seems, however, to have proved fatal to the Massachusetts Bill, which was finally withdrawn at the end of March. In Pennsylvania the Bill for the Registration of Nurses proposed by the graduate Nurses' Association has been defeated in the Senate of that State. The opposition appears to have come chiefly from the authorities of hospitals for the insane, and special hospitals, as in the case of Massachusetts. The opposition was no doubt largely affected by the influence of Dr. S. Weir Mitchell, who is reported to be very much opposed to the Pennsylvania Bill. The rejection of these two Bills would seem to indicate that as the subject of State registration is becoming better understood in the United States, public opinion is growing more and more opposed to legislation on the relatively narrow lines which have governed the control of State registration, as enacted in New York, New Jersey, Virginia, North Carolina and Maryland.

### A Society for Promoting Higher Education.

It is interesting to note, though there is no connection between the two movements, that in Massachusetts Dr. Richard Cabot, who is widely known and respected by nurses, has succeeded in establishing a New England Association for the Education of the Nurse, of which he has become the first president. The inception of the new society is attributed to Dr. Worcester of the Waltham Training School, and the scheme as modified omits all reference to any system of nurse registration. The Society will be managed by a committee of twenty, and a considerable number of nurses have already joined the new society. Its chief object is educational, Dr. Cabot being the foremost advocate of the higher education of nurses, and he is strongly opposed to the employment of pupils in training as a means of revenue for the hospitals. So far as it is possible to gauge American opinion it would appear that the New England Association for the Education of the Nurse represents the rebellion of public opinion against the exclusion of members of the medical profession and trustees or representatives of the hospital training schools from the State registration boards, which the more advanced leaders of the nurses in the United States are doing their utmost to secure.

### Novel Provisions in Registration Acts.

In Colorado an Act has been passed for the State Registration of Nurses. The State Board is to consist of five members, all of whom are to be trained nurses, and they are to elect from amongst themselves a president and a secretary, the latter of whom is also to be the treasurer. Each applicant for registration has to pay a fee of £2. The Act permits any nurses engaged in nursing who show to the satisfaction of the Board they were so engaged prior to the year 1901, having given 18 months' general training, to register, providing they make application before April 1906. The Act makes it unlawful after that date for any nurse to practise nursing as a trained, graduate, or registered nurse without a certificate from the State Board of Examiners. But any person may nurse the sick for hire who does not in any way assume to practise as a trained, graduate, or registered nurse. Former acts have only penalised those who wrongly used the title Registered Nurse, and it remains to be seen how far it will be possible to work the Colorado Act in view of the provision just quoted. It is noteworthy, too, that this Colorado Act is not to apply to nurses who have served as such in the army of the United States in the civil way or the Spanish-American war. In California, an Act for the Registration of Nurses, dated March 21st last, states its object to be to promote the better education of practitioners of nursing the sick. This Act entrusts the duty of examinations and the maintenance of a register, with the enforcement of discipline, to the Board of Regents of the University of California. The fees are only £1, but the certificate of registration is to be void at the end of three years; a new certificate may, however, be issued to each holder upon a further payment of 5s. After January 1st, 1908, no person will be eligible for examination or registration as a trained nurse who is not a graduate of a training-school approved by the Board of Regents of the University of California. Such training-school must be attached to a general hospital, and must insist upon each nurse undergoing a course of three years' training in its hospital, or at least one year's such training there in addition to a two years' course in the training school of a special hospital. After January 1, 1910, no person will be eligible for examination or registration unless he or she furnishes satisfactory evidence of having substantially completed the course of studies pursued in the grammar schools of the State of California, or an equivalent course. The latter is a novel provision, and emphasises the fact that the tendency in the United States is to stiffen up the general education of nurses, and to make all candidates for training produce evidence of having attained a definite standard in this respect.

### A Private Nurse's Earnings.

It is interesting to note the earning power of a private nurse in the larger cities of the United States. In central New York, for instance, private nurses are paid $20 (£4) per week. A really capable and industrious woman, who devotes herself to her work, it is stated, may be reasonably sure of employment for 45 weeks in each year. A nurse may thus earn $900, or upwards of £180, and have seven weeks free from duty. The cost of living in New York is fairly high, but as a private nurse when on duty has no expenses for her maintenance to pay, her total outlay each year need not exceed $400, or some £80 a year It will thus be seen that a good nurse in full employment in an American city has a considerable margin for saving, and that her net earnings are, in reality, higher than those which prevail in this country.

### The Higher Training of Midwives.

### OPENING OF A HOSPITAL AT WOOLWICH.

A NEW Hospital for Mothers and Babies, with which is connected a training school for district midwives, was opened at Wood Street, Woolwich, on Thursday last week by Princess Christian. Amongst those present were the Countess of Stamford, the Marchioness of Ailsa, Lady Maurice, Lady Donaldson, and the Dean of St. Paul's. Her Royal Highness was received by Canon Escreet, rector of Woolwich, and accepted a bouquet from the Mayoress, after which an address was read. The following is the substance:—

The profession of midwifery among the poorer classes has been, up to the present, almost monopolised by a large number of supremely ignorant women, with the result that the mortality among their patients from septic infection and other preventible causes, has been very high. At present the country is most inadequately furnished with trained women to succeed them. Our excellent maternity hospitals are training many pupils yearly, but by far the greater number of these pupils are proposing to devote themselves to private nursing and leave the district problem untouched. This work of district midwifery is a very unpopular one among educated women, and more especially among nurses. It is not difficult to realise some of the causes of this unpopularity. The training fees are high, even when the course can be obtained in a very few months—the responsibilities too great to be lightly undertaken by conscientious women on the short training that suffices for private nurses who will only be called on to work under a doctor, and the fees received frcm the poor are naturally very much lower than those taken by the latter class. It has been therefore the earnest wish of the Council for the Promotion of the Higher Training of Midwives to provide a school where the training shall be of longer duration, and especially adapted to those working in district, and where the fees shall be moderate if existent. In starting this it is hoped that a beginning at least is about to be made in the right direction. It is intended that the pupils in this training school shall be of an educated class, and that they shall have received at least one year's previous training in the wards of a general hospital. Further, that until the home is in a position to offer a free training, a fee of £20 shall be charged for a course of from six to twelve months, according to the requirements of each individual pupil, and it is hoped that scholarships for this amount may be offered by the various County Councils.

After Princess Christian had declared the home open, a vote of thanks was proposed by Dr. Cullingworth, who said that the interest taken by her Royal Highness in social questions was well known, and the fact that she always made careful inquiry before she associated herself with any undertaking, spoke well for the claims of the home she had just opened. He also expressed his own sympathy with the objects of the new home.

The blessing having been given by the Dean of St. Paul's, the visitors were shown all over the building, which consists of two houses standing on elevated ground, which have been thrown into one. It was at first the idea of Miss Alice Gregory, who is the honorary secretary, to wait for the development of her scheme until she could afford to build a home, but Princess Christian strongly advised a start being made in an adapted house, and the institution at Woolwich is the outcome of her suggestion. Through uniting the two entrances by means of a large arch a fairly spacious hall has been obtained. On one side of this hall is the nurses' dining-room; on the other a large ward, at present unfurnished, but which is called "Hope Ward," in a prophetic spirit. It will ultimately make an ideal room, as it runs the whole way through the house, and has a large window going on to a tiled balcony, upon which the convalescent mothers can lie out and grow strong. The flowering trees and garden space are a decided boon, but Miss Gregory fears that, unless some generous friend comes to the rescue and enables the council to purchase, fresh houses will shortly cover the ground, as it is to be let for building.

On the first floor there are two wards, one containing three beds and another two. All the arrangements are admirable, the most striking being the beds. These are painted white, and are covered with pretty blue and white cotton counterpanes. At the foot of the bed are two strong iron supports bending towards each other, and ending in a hook. From this is swung a sweet little cot, of white enamelled iron, covered in white chintz, the whole having been manufactured to order by a London firm from the matron's design. The bedclothes were made by the members of the Ladies' Guild. Beside each bed stands a glass table, with a drawer beneath to act as a locker for the patient's personal belongings. The drawer, as well as the top of the table, can be easily disinfected when required. Though no pictures are allowed in the rooms, the delicate colouring of the pale-green walls, the pretty pink and white flowered screens, the dainty window curtains, and the cosy American rocking chairs and cushions, all tend to give an appearance of refined comfort.

Upstairs are the pupils' bedrooms—each has a separate room—nicely complete, with fumed oak furniture, and also a room for a private patient, whose fees vary according to her powers of contribution. The matron's bedroom is approached through the dispensary—a well-stocked little room—and Miss Gregory's office on the corresponding side of the other house, acts as an ante-room to her own sleeping apartment. Owing to the union of the two houses everything is in duplicate—two bathrooms, two lavatories, for instance. In the case of the latter, bowls, basins, etc., are washed in one, whilst all soiled crockery, such as bed-pans, are sent to the other side of the house, the arrangements for these being such that they can be thoroughly cleansed without the nurse touching them with her hands. The labour room is quite up to date. The walls are made of Ripolin, which is less expensive than white tiles and is said to be equally efficacious; all taps are turned on by foot pressure; and specially large glass bowls in which a nurse can plunge her arms above the elbow and sterilise them at once are provided. There is also a tiny chapel at the top of the house where morning and evening prayers are said, and an isolation ward for infectious cases.

The staff consists of the matron, Mrs. Parnell, who was trained at the New Hospital for Women and at the British Lying-in Hospital. For the past eleven years she has been matron of the Memorial Cottage Hospital at Paulton, between Bristol and Bath, to which an out-patient maternity branch was attached. She is also a certified midwife, and holds the Apothecaries' Hall certificate, so that she is able to undertake the dispensing of the new Home. Mrs. Parnell, together with Miss Gregory and another sister with two years' general training, attends to the patients and instructs the pupils. It may be added with respect to the latter, that though the £20 fee is only supposed to cover six months' training, if it be found necessary the teaching will be increased to a year without further fee. The pupils must provide themselves with indoor and outdoor simple uniform, and before leaving pass the examination of the Central Midwives Board. There is no specified age, the matron dealing with each application on its own merits. The pupils receive thorough instruction at the Home before they go into the district, and they are then accompanied by one of the sisters to show them how to put their theory into good practice with limited appliances.

### Select Committee of the House of Commons on Nursing.

### Evidence of Mrs. Bedford Fenwick.

The Select Committee of the House of Commons on Nursing met on Thursday, last week. Mrs. Bedford Fenwick was called as first witness. She began by stating that she was practically in agreement with what had before been said by Miss Stewart, Miss Huxley, and Miss Forrest. After having summarised the conditions under which registration was carried on in some of the Colonies and the United States, beginning in the Cape of Good Hope as early as 1891, she proceeded to state that while none of the existing registration laws satisfied the needs of nurses in England, she felt that in New Zealand, where the matter was in the hands of the Government, and registration was conducted by Government officials, they had approached nearest to the ideal, and the effect was good from every point of view.

Mrs. Fenwick proceeded to say that she felt strongly that there should be an educational standard fixed, and that the education of a nurse should be placed on a sound basis, and she judged that this could only be done through a central board. She held that through the want of some such standard many training schools for nurses were in the hands of incompetent women, whose chief recommendation, perhaps, was their housekeeping capabilities, and not their power of imparting knowledge to others, or of keeping good discipline. She was sure that an advance in the standard attained by nurses would be one of the many benefits arising from State registration. The curriculum defined for the education of a nurse should be the minimum necessary; the examination would merely be a test at the conclusion of the training. At present bad nurses competed with good, and there was a growing sense of injustice among nurses. She would like to urge as strongly as she could that the question of justice to nurses should receive due consideration; they were a valuable national asset, and promoted the well-being of the people. With regard to the suggestion of a former witness, that registration would induce nurses to pretend to a scientific knowledge which they did not possess, and to assume the responsibilities of the medical faculty, she emphatically denied the imputation, and, questioned by the Chairman, she replied that she thought sufficient proof might be adduced against this by the fact that the best nurses were on excellent terms with the medical faculty, and that any such assumption would be due to ignorance, and want of self-discipline and knowledge of nursing etiquette.

The Chairman having referred to the suggestion which had been made that nurses would be unable to pay a fee sufficiently large to cover the expenses of the Central Board, Mrs. Fenwick expressed the opinion that nurses would be glad to pay five guineas for the privilege of being registered, three guineas for the examination, and two guineas for registration. Questioned by the Chairman on the supposed danger in hall-marking a nurse once for all by registration, she rejoined that she thought there might be some system of supervision and inquiry, and that a nurse might be struck off the register after investigations by a sub-committee appointed for the purpose. But she believed that there would not be any very large number of cases of deterioration when once the education of nurses had been defined. As to the suggestion that registration would make it difficult for poor people to obtain good nursing, she did not think that such would be the result. It had not been so in the case of the registration of medical men. With regard to the registration of nursing homes, as she had herself managed one for seven years, she could speak with some knowledge, and she was strongly in favour of them being registered. She thought, however, that this matter should be in the hands of Local Authorities, and altogether apart from the registration of nurses.

With respect to the alleged number of bad nurses, Mrs. Fenwick did not think that the number was overstated, and handed in a summary of a hundred cases which had come to her notice of the trial of nurses for criminal and other offences. Asked by a member of the Committee whether she suggested that the Central Board could go behind the decisions of the Courts, she replied in the negative, but she said that the Board would prevent the nursing associations from sending out undesirable nurses insufficiently trained. Cross-examined later by various members of the Committee, Mrs. Fenwick admitted that in a large number of cases the result of the trial was not known. When it was pointed out that, considering the number of nurses, the percentage of bad nurses was exceedingly small supposing her list to be exhaustive, she replied that she felt sure her list was by no means exhaustive.

Mrs. Fenwick did not anticipate that registration would stereotype training and stultify progress, as had been suggested. With regard to the supposed hardship in the insistence on a three years' course, she quoted statistics to show that, with but few exceptions, a three years' course was already insisted on by most hospitals. She suggested that there should be a two years' term of grace, in order that existing nurses might not suffer by the Act.

Asked whether she was in favour of lay representatives on the Board, she said that she had no objection to lay persons being on the Board, though the Board should mainly consist of medical men and nurses.

Mrs. Fenwick stated that in her experience as honorary superintendent of the Registered Nurses' Society, she had frequently found that no registers were kept at some hospitals, and she had experienced great difficulty in tracing a nurse's record. She thought that registration would have great moral weight in preventing those who were not really qualified to be nurses from practising as such.

### Evidence of Miss Dock.

Miss L. L. Dock next gave evidence. She explained that she had no experience whatever of nursing in this country, and was only prepared to give the Committee information about the conditions prevailing in the United States. She was trained at the Bellevue Hospital, New York, and had had eight years' district nursing in various parts of America, chiefly New York, Chicago, and Baltimore. Her evidence was mainly statistical, or traversed the ground with regard to existing legislation in America, which had already been dealt with by Mrs. Bedford Fenwick and it was therefore, at the request of the Chairman, handed in mostly unread. Miss Dock, however, stated that the minimum of two years' training as required by legislation was gradually being voluntarily raised to three through the efforts of matrons and others, and she herself thought that three years should be the minimum. She said that registration was only permissive in the States, but that in her opinion it should be compulsory. The promoters of registration, however, had not dared yet to press for this point. The number of nurses registered in Virginia was 400, and in New York 1,200. The fee for registration was £1 in some States, £2 in others. There was in some States a partial revision of the register, and nurses paid about 4s. for the renewal of their licenses. The fee for registration included the examination, and Miss Dock stated that so far the fees had covered all the expenses of machinery, and she did not anticipate that they would be raised. Asked whether there had been a shortage of nurses owing to registration, Miss Dock said that though the numbers had in some parts decreased, the quality had certainly risen. She expressed herself more than satisfied with the result so far, and felt that though there were difficulties, they were not insuperable.

The Committee sat again on Tuesday morning.

EVIDENCE OF MR. UPTON.

The evidence of Mr. J. R. Upton, solicitor to the Society of Apothecaries, was first taken. It only occupied the Committee but a short time, Mr. Upton's main point of argument being that in the opinion of his Society the registration of nurses would lead to their attempting to compete with doctors. Reminded by the Chairman that a similar argument was used when the Midwives Bill was before Parliament, he admitted that that was so, but that it was yet too early to predict the resul's of the Act, and that the sphere of a midwife was more limited than that of a nurse. The opinion he expressed as to the competition that would arise between nurse and doctor was not, however, he said, based on any personal knowledge of cases where a better qualified nurse had presumed on her position. Questioned by a member of the Committee, he said that he thought it would be desirable to have institutions registered. He claimed on behalf of his Society that as they were represented on the Midwives Board they might be represented on the Registration Board should the Bill become law.

EVIDENCE OF SIR JAMES CRICHTON BROWNE.

Sir James Crichton Browne gave evidence at length and with much detail. He stated that, though there had been an enormous improvement in the condition of nursing of late years, there were still a great many incompetent nurses and a great want of uniformity, and that a scheme, such as was contemplated, would, in his opinion, winnow out the undesirables; that the qualifications demanded should be the minimum necessary, but quite adequate, and that, as was the case with the medical faculty, nurses would no doubt proceed to further qualify themselves. Entrance into the ranks of nurses should be by the "one-portal" system. As to the question of the character of a nurse the matron of the hospital would have ample opportunity of observing her qualifications and moral character during the three years to be spent at the hospital, as laid down in the Bill of the Royal British Nurses' Association. The association had already 2,600 registered nurses and he himself was constantly in the habit of turning to this register when a nurse applied to him; the public would learn to do the same if a State Register were kept. He thought that the 80,000 nurses of the general census must certainly have included the 11,000 asylum nurses, all probationers, a large number of casual and occasional nurses, and probably many children's nurses. It was extremely difficult to form an estimate of the number of nurses. He was inclined to think that at most nurses would number 60,000, and that 45,000 would be nearer the truth, including probationers. Of these not more than, say, 20,000 would come on the register at once, and from a computation of the average nursing life of a woman he would judge there would be some 3,000 who would leave the register per annum. He admitted that 2,600 (the number registered by the British Nurses' Association) was but a small percentage of the whole, but the Association was merely voluntary, and was open to the adverse influence of matrons and others who greatly swayed the nurses. He did not hold that opposition would be continued against a State register, any more than it had been continued against the registration of doctors. There should be recognised training schools, so that no hospital where training was given would be able to fall below a certain level, and the Board would need to send inspectors round to see that the work was being properly carried out. His idea was that the nurses should send the certificates they had gained at an institution to the Board some time before the examination, and that the examination should be held at different centres throughout the country. Asked by the chairman if he did not think, as had been contended, that recognition of training-schools would be a sufficient and much easier solution of the question, Sir James replied that he did not think it would at all meet the case. When once a nurse had left the institution she was no longer connected with it, and under no supervision.

As regards recognition of training schools, Sir James said he would advocate the recognition of no hospital under 40 beds, and would require three years' training, two of which at least should be spent in a general hospital. Asked his opinion on the question raised as to the competition between the nurse and the doctor, he replied that he had always found it was the ill-educated nurse who did not know her place. He thought that if culpable negligence or unskilfulness were proved against a nurse, her certificate should be suspended, just as in the case of captains of vessels. As to the proof of negligence, he was of opinion that the evidence should be privileged, and that the doctor's evidence should be taken, but with regard to any evidence by the patient or the patient's friends, he held that such evidence should not be privileged.

As to the attestation of character he was of opinion that this would be done sufficiently once for all by the certificate from the hospital, based on three years' observation, and the Board would also have some opportunity of judging character at the *viva voce* examination, it being understood, of course, that the Board could refuse to register any nurse. He did not think that any system of "espionage" was desirable. He would, however, require each nurse—say, every two years —to make a return to the Board, giving her address and stating whether she was still nursing. As to the fees to be paid he thought that three guineas—two for registration and one for examination—would be a reasonable charge with, perhaps, a charge of 2s. 6d. for revising the entry in the register. This would give the Board sufficient money for its purposes, especially as the members of the Board, being mainly matrons and sisters, would not need to be paid so highly as physicians. He was quite sure that a nurse could meet the charge, as from his connection with the Royal Chartered Nurses' Association, which placed nurses, allowing them all moneys earned minus 7½ per cent., he could state that their nurses made on an average £96 a year. This was different, of course, from the rate of earnings through an ordinary agency.

As to the question of having two classes of registered nurses, Sir James said that it would be advisable to have a second class called "nurse-helps" which should comprise such nurses as those who were recognised under the County Councils as assistants to district nurses. If a list of these were kept, it would better their position, and they might be placed on the list, say, after six months' training in a hospital and six months' work under a district nurse; he did not advocate any examination for such nurses. He thought that nursing homes should be registered, and this work put into the hands of County Councils, a home being taken to mean a house where more than two sick persons were received. As to mental nurses, he considered that the certificate of the Medical Psychological Association should be accepted by the Board, and that such nurses should then be added to the Register in a separate list.

Questioned by Dr. Hutchinson, he conceded that the public were sufficiently protected in the case of what formed the largest body of nurses, namely, the staff of nurses at hospitals and such institutions. He would not admit the suggestion of Dr. Hutchinson that a register kept by County Councils of the institutions which should be recognised as training schools would meet the case of the further class of nurses sent out by institutions. He felt sure that a Central Board was necessary on account of the present variation in standard. He considered the suggestion of Mr. Sydney Holland that nurses should be struck off the register when they reached a certain age, most unfair; and he would not strike off a nurse, unless culpable negligence were proven against her, except at her own desire, even though it was evident by her returns every two years that she was no longer in nursing practice. He would leave it in the hands of the public to make the necessary inquiries as to her last employer. As to the possibility of the supply of efficient training schools being insufficient, he was of opinion that county hospitals could take more probationers, and that workhouse infirmaries could be more utilised than they were at present.

In answer to Sir John Batty Tuke, Sir James said he thought that the examination should be practical as well as theoretical, and that such an examination was certainly necessary, in addition to the examinations held at the various hospitals, in order to secure that uniformity which was so lacking.

### A Book and its Story.

### PROBATION IN A PROVINCIAL HOSPITAL.*

Ellis Deane has apparently written her book with the intention of bringing some aspects of hospital training before intending probationers, that they may see that it is actually very unlike the ideal that exists in the imagination of most girls who are thinking of nursing as a career.

For the sake of the nursing profession generally we hope that the very unattractive picture given of the relations between the staff nurses and their subordinates, in "A Raw Probationer," is either an exaggerated one or represents a small minority of institutions in which personal feeling, instead of the general well-being of the probationers, regulates the conduct of those in authority over them.

The story opens with rather a sad picture. Joan Aldis, the heroine, is saying good-bye to Dr. Arnold, at whose recommendation she has been taken by the matron of Redburn Infirmary as probationer. Poor little Joan! This is what she says for herself of her past life and the present moment, which, in spite of her sorrow, she faces bravely.

"Just six weeks ago to-day I knew that my father had gone down with his ship within three days of home—losing his life, it was said, in a brave attempt to save others. I had been unusually full of joy that day, as I always was when his home-coming drew nigh, singing snatches of happy songs about the little home on the cliff that he liked me to keep for him with the help of, and guardianship of, old Nancy, who had been with my mother also before she died. And then—the news came. It did not seem to matter, after that, that I knew there would be nothing to live on—that in his happy-go-lucky way father had been forgetting his insurance premiums a little too long; nothing mattered beside the thought that he whom I loved so dearly, and been so proud of in his strong, handsome manhood, who had represented all the world to me since my mother's death, four years ago, would never come again. Dear Dr. Arnold! What should I have done but for him the last few dreadful weeks I do not know! He was my father's closest friend for years, and I used to wonder what it was in him that my father—and my mother too in her lifetime—could see to think so much of, but I shall never wonder again." Dr. Arnold had offered Joan a home if she would stay with him, but this she had declined. "If I had to work, it might be as well first as last; and so, when he saw that I was determined, he helped me to the one thing I could think of as a means of livelihood—hospital nursing. I do not know what put this into my head, except that it had always been there in a hazy kind of way ever since my school days, when one of the teachers had made Florence Nightingale the subject of special notice." Dr. Arnold had tried to dissuade Joan from carrying out her intention. He knew that her age was against her—she was nineteen—and a few years later she would be eligible if she still wished to become a nurse. But, as her story shows, when Joan had made up her mind she carried out, when possible, her ideas, and bore, not always with patience, but without faltering, the result. At parting Dr. Arnold had given her some injunctions as to her demeanour towards his friend, Miss Warner, the matron at Redburn Infirmary. Miss Warner had taken Joan as a probationer at once on hearing the circumstances of her case, and Dr. Arnold's personal knowledge of her. "There is one thing," says Dr. Arnold somewhat hesitatingly and very tenderly, "one thing I must not forget to warn you about—be most careful to propitiate Miss Warner. She will be prepared to like you, I hope, for my sake, but she is rather a peculiar woman, and I fancy could make it unpleasant for any one she did not take to. She is bound to like you ultimately, but, just at first, I put you on your guard, my dear, for I know how much it is in the matron's power to do for you, or otherwise. . . . And now, my dear, good-bye. You must write to me regularly, and tell me exactly how you get on—no glossing over disagreeables, remember. And above all, always think of me if you want help." Not every probationer in real life starts out fortified with the advice given by Dr. Arnold to his protégée. Opinions may differ as to the wisdom of prejudicing the mind of an inexperienced girl against Miss Warner. Surely if a matron is a woman of discernment such advice was quite unnecessary. She would be able to judge for herself, and to make allowances for youth. She would not descend to, nor likely to be influenced by, the bearing of a probationer towards herself. Nor would any girl with sense be likely to need such a warning; self-respect, respect for those in authority, and a realisation of her own ignorance, would surely make it unnecessary. So little Joan starts on her journey, and very hard indeed must be the reader who is not moved by the forlorn little figure travelling from the green hills and fields of the south to the grime and darkness of the smoky town of Redburn. "I stand with my head out of the carriage window as long as I can see Dr. Arnold, then I sink back into my corner and still, with my face to the window, blink out of my eyes the tears that have gathered there. I am not going to cry and make a spectacle of myself for the strangers in the carriage to pity. Besides, I have cried so much, I do not think I shall ever cry again. And all the crying in the world would not put me back where I was six weeks ago. Just six weeks ago to-day I knew that my father had gone down with his ship within three days of home."

Joan's first interview with Miss Warner does not dispel the fears which Dr. Arnold's advice had aroused. Upon her arrival at the infirmary she is handed over to a "dignified, kindly-faced woman in nurse's uniform." This is Nurse Walton, to whose ward Joan is to be appointed. She expresses her surprise at Joan's youthfulness. "Excuse me, but you are very young, are you not?" "I am nineteen," I tell her, with all the dignity I can muster, "and I feel much older." "It is younger than nurses usually begin," she replies. Then Joan is summoned into the presence of Miss Warner, and her verdict is even less encouraging. "Are you Miss Joan Aldis, the girl Dr. Arnold has recommended to me for the nursing staff?" "Yes, madam." "Then, all I can say is, Dr. Arnold must have taken leave of his senses. Why, goodness, child . . . you don't look seventeen. It is the most preposterous thing I ever heard of! . . . You poor child! You haven't an idea of what you are talking about. The place where a hand is wanted is the men's surgical ward, and the cases you would frequently have to attend to would frighten you out of your wits." Joan is urged to give up the idea of being a nurse and to try something else. But she perseveres in her intention, and, in spite of a period of probation, made specially trying by some of those with whom she comes into immediate contact, she gives satisfaction generally, and falls ill through undermining her strength, and becomes an easy prey to typhoid fever. An ideal character is introduced in Mary Winston. In her, sweetness and strength are combined equally, and charming looks go to make a perfect picture of an ideal nurse. "A Raw Probationer" will naturally interest hospital readers, and, apart from the nursing experiences, there is enough romance to make it attractive to everyone. The home life at Daleswood Manor is particularly well described, and the experiences of Joan in Egypt make plenty of diversion.

* A Raw Probationer. By Ellis Deane. (Digby, Long, and Co. 6s.)

### Everybody's Opinion.

### REGISTRATION FEES.

"A Nurse Without Private Income" writes: I am not a keen partisan of registration or the reverse, believing that if registration comes we shall be very much in the same position as we are now, with both black and white sheep in the fold. But before the iron hand of the law grips us I do hope that someone with a kindly sympathy for ill-filled purses will come to our rescue. Mrs. Bedford Fenwick's suggestion that "we should be glad to pay five guineas for the privilege of being registered" will be by no means endorsed by the majority of nurses, who find it hard enough now to pay for dress, holiday, and Pension Fund subscription. To be obliged when we have finished our training—often at 28 or 29—to pay another £5 5s. before we can begin to earn a penny is too much of a hardship. Moreover, if £1, or at most £2, is sufficient for the purpose in America, as Miss Dock stated, why more than double the charge here, where, on the whole, nurses are not so well paid?

### THE REGISTRATION OF NURSES' UNIFORMS.

Miss Dorothy A. Davey writes: It seems a pity that you should proclaim such adverse criticism of my proposal and in such an emphatic manner, particularly as her Royal Highness Princess Christian approves of it and has graciously commanded that the matter be placed before the Association of which she is president at their next meeting. Several members of Parliament have also sent kind letters of approval and congratulation, which at least proves to me that the scheme is quite worthy of further consideration. In justice to my scheme will you kindly recognise the fact that at most there are only three designs—based on the same model—the remaining differences being in the colouring of the enamel. The last clause of your note I need not dwell upon, as it is only necessary for the general public to realise that a Government-protected badge is worn by all certificated nurses. Anyone wishing to know the reason of the variations in colour, etc., need only inquire of any nurse wearing her badge.

### THE ABUSE OF NURSING ASSOCIATIONS.

"The Honorary Secretary of the Carlisle District Nursing Association" writes: It seems only fair to our superintendent and the district nurses to write a word of explanation regarding the remarks headed "The Abuse of Nursing Associations" in The Hospital of May 6, which refers to a point raised by our late honorary physician. His remarks were prompted by the difficulty our superintendent has in making the public understand that our district nurses are not for those who can afford to engage a private nurse, but for the sick poor who are unable to pay, but who give a donation when they can afford it. We do not believe that our association has suffered, as the executive committee is in touch with all the cases; also the number of cases and visits paid is a record for our association. Our subscribers, we believe, have confidence in our work. Another point which has to be taken into consideration is the great number of our patients whose husbands or fathers were out of work during the winter.

### NURSES AND MONEY-MAKING INSTITUTIONS.

"A Sympathiser" writes: While the question of State registration is on the tapis, could not something be done to abolish the sweating system in the nursing profession and to enforce and make the co-operation system more general. I have seen it stated in a nursing journal "that imperfectly trained nurses are often compelled, as a last resource, to work for some money-making institution which farms them out, reaping a rich harvest for services for which it pays them barely a living wage." But is it not the case that numerous nurses who are thoroughly well trained and experienced in all branches of their profession are compelled—owing to competition, etc., and the small number of co-operations to work for these money-making institutions, giving the best of their years, health and strength in anxious toil for one-third of their earnings? I know of instances where these homes and institutions are kept afloat, not by the patients who enter them, but by the fees earned by the hardworking nurses. In many cases these nurses, before joining, and sometimes after, are presented with an imposing-looking document to sign which threatens them with a penalty of £50 should they attempt to work for their own fees within a radius of, sometimes, 20 miles. Of course the document is not legally binding, but many nurses are not aware of this and therefore when a chance of improving their position arises, are deterred from taking advantage of it by the fear of this £50 fine.

### AN OLD DODGE.

"Once Bitten Twice Shy" writes: In the interests of the public, I beg to draw your attention to a fraud of which I was made the victim last week. Having advertised in your paper for a post as "Nurse-Companion," a lady called on me on Saturday afternoon, representing herself to be the wife of a doctor, giving name and address at St. Albans. She said her sister required a lady to help her in the management of a home for invalid and decayed gentlewomen, giving name and address near Leeds, and naming salary, also giving details of the post and duties required. After a certain amount of conversation, inquiries about references, etc., she seemed to think I was suitable, and arranged an interview with her sister for Monday, pending letter or telegram to settle details. She then casually remarked that her daughter and a friend who had accompanied her to town had gone to Hampton Court, and taken her purse with them, which she did not discover till they had left her, leaving her without funds for any necessary expenses in getting back. She also observed that they were giving a small dinner party that night, and she had promised to take back the fish with her. Having no reason to doubt her story I offered to help her out of the difficulty with a small loan, and she ended by accepting a sovereign. I have neither seen nor heard anything of the "lady" since, and on looking up a "Medical Directory" find that there are no such addresses as she gave me at either Leeds or St. Albans. She was a person of medium height, rather stoutly built, middle-aged, fresh colour, dark hair, and wore glasses, quietly dressed in black, with either small hat or toque, nothing at all noticeable in any way about her.

[We have had other complaints to the same effect.—Ed. The Hospital.]

### PROPOSED PETITION TO PARLIAMENT.

"An American-trained Nurse" writes: While reading the voices for and against the Higher Education Scheme, I was specially struck with one idea, viz., that nurses are capable and competent to manage their own business. But, on turning over the pages, there is a cry for Government to investigate and issue badges to individual nurses. Poor Government! Now, as the matrons do not appear to initiate any step in the suppression of the abuse of our uniform, it seems to me that nurses should take it up. Perhaps the Hostel Company would be the most likely medium, as all kinds and classes and quantities of nurses pass through the Nurses' Hostel in a year, and Miss Wood takes a tremendous interest in nurses and nursing. Why could not a petition be drawn up and presented in Parliament prohibiting any person from wearing any part of a hospital nurse's uniform? Whether the certificate is for one year or three, so long as it is granted it ought to be recognised, if such certificate were granted before the year 1901. Nurses with one year's training and "brains" probably learn more in that time than their sisters with three years' and a "cerebral cavity." Besides, the former worked harder for the hospitals when fewer nurses were available, or she paid £1 1s. a week toward the hospital funds; therefore the hospitals, which make no provision for supplementing her training and granting her the up-to-date certificate, should be careful how they crush her out of the nursing profession, Let the full force of their energy be thrown upon nursing homes and the so-called training of nurses in them. Neither probationers, nor servants nor lady-helps, in nursing homes should be allowed to wear the uniform. Only nurses with one to three years' training in hospitals or recognised infirmaries supporting resident medical officers should be allowed to sign the petition. Of course, a certain number of signatures would be necessary, though it need not be a six-mile petition, like one at present in the House, but short enough to allow plenty of time for consideration.

### Appointments.

Bedford Workhouse Infirmary.—Miss Martha Louisa Taylor has been appointed superintendent nurse. She was trained at Bristol Union Infirmary, where she has since been assistant nurse and charge nurse. She has also been superintendent nurse at Romsey Union Infirmary.

Clayton Vale Hospital, Newton Heath, Manchester.—Miss A. G. Lett has been appointed staff nurse. She was trained at St. Luke's Hospital, Halifax, and the Isolation Hospital, East Ham, E.

Cottage Hospital, Cockermouth.—Miss E. Linton has been appointed matron. She was trained at the Burton-on-Trent Infirmary, and has since been charge-nurse at the Birmingham and Midland Ear and Throat Hospital; nurse-matron at the Cottage Hospital, Liskeard; holiday sister at the Bridgwater Infirmary; and senior nurse at the Eltham Cottage Hospital. She has also done private nursing in Reading and Newcastle-on-Tyne.

Hospital for Women and Children, Lupus Street, Westminster.—Miss Mary E. P. Cornish has been appointed matron and midwife for the district connected with the hospital. She was trained at the Children's Cottage Hospital, Coldash, near Newbury, and has been assistant nurse at the Western Fever Hospital, Fulham, and on the private staff of the Warneford House Nursing Association, Leamington. She is registered under the Central Midwives Board.

Infants' Hospital, Denning Road, Hampstead.—Miss A. E. Howard has been appointed sister. She was trained at the Queen's Hospital, Birmingham, served in South Africa as a member of the Army Nursing Service Reserve, and has since been sister of the male wards, and theatre sister at the Royal Cornwall Infirmary, Truro.

Jenny Lind Hospital for Children, Norwich.—Miss R. Butler has been appointed staff nurse. She was trained at the Victoria Hospital, Hull.

Leeds Union Infirmary.—Miss Edith Whittaker has been appointed home sister. She was trained at Chorlton Union Infirmary, Manchester, where she has since been sister.

Lincoln County Hospital.—Miss E. Marchant has been appointed sister. She was trained at the Sheffield Royal Hospital.

Llandudno Isolation Hospital.—Miss E. P. Foster has been appointed staff nurse. She was trained at Plaistow Fever Hospital. She has since been charge nurse at the City Hospital, Birmingham, and staff nurse at the City Hospital, Leeds.

Notts Consumption Sanatorium, Sherwood Forest.—Miss Annie Broadbent has been appointed matron. She was trained at the Royal Infirmary, Bradford, where she has since been night superintendent, housekeeper, and home sister successively; she has also done private nursing.

Plumstead Union Infirmary.—Miss Sarah H. P. Leslie has been appointed ward sister. She was trained at St. Bartholomew's Hospital, London, and has since been superintendent nurse at the Southwark Union Infirmary; she has also been nursing sister in the Indian Army Service.

Royal Bath Hospital, Harrogate.—Miss Marie Willis has been appointed staff nurse. She was trained at the Hospital and Dispensary, Rotherham, Yorkshire.

Royal Hospital for Diseases of the Chest, London.—Miss Mary F. May has been appointed matron. She was trained at St. Bartholomew's Hospital, London, and has since been lady superintendent of the Grosvenor Hospital for Women, London, acting matron of the East Suffolk and Ipswich Hospital, and matron of Bridgwater Infirmary.

Royal United Hospital, Bath.—Miss Mary Brett has been appointed night superintendent. She was trained at the Royal Hants County Hospital, Winchester, and has since been attached to the private nursing staff of that institution. She has also been nurse at the New Hospital for Women, Euston Road, London, and has taken matron's holiday duties at Farnham Isolation Hospital. She holds the certificate of the City of London Lying-in Hospital for maternity nursing, that of the Central Midwives Board for midwifery, and a certificate for massage and electricity.

Shoreditch Infirmary, London, N.—Miss Emilie Stephenson has been appointed ward sister. She was trained at Leeds General Infirmary, and has since been staff nurse at St. Peter's Hospital, Covent Garden, London. She has also done private nursing.

Southwark Infirmary.—Miss Annie Elise Hartmann has been appointed sister. She was trained at Southwark Infirmary.

Stepping Hill Hospital, Stockport.—Miss Jane Jeffery has been appointed matron. She was trained at the Sick Children's Hospital, Edinburgh; the Middlesex Hospital, London; and the Battersea branch of the Clapham School for Midwives. She has since been superintendent nurse at Newton Abbot Workhouse Infirmary and at Cheltenham Workhouse Infirmary, matron of the Cottage Hospital, Exmouth, and home sister at the Workhouse Infirmary, Stockport. She is registered under the Central Midwives Board.

Strood Workhouse Infirmary.—Miss Lucy Creemer has been appointed superintendent nurse. She was trained at Camberwell Infirmary, where she has since been staff nurse, ward sister, and night superintendent. She was also nurse at Cane Hill Asylum for 18 months.

Willesborough Infectious Hospital.—Miss Jessie Allan has been appointed nurse-matron. She was trained at University College Hospital, London, and was subsequently attached to the private staff. She has also been sister of the enteric wards in the Corporation Sanatorium, Lancaster, and served as nursing sister during the war in South Africa.

Willesden Workhouse Infirmary.—Miss Mary Jolly has been appointed charge nurse. She was trained at Bethnal Green Infirmary. She has since been nurse at the Florence Nightingale Infectious Hospital, Bury, Lancashire.

### Novelties for Nurses.

(By Our Shopping Correspondent.)

### WHERE TO FURNISH.

Messrs. Oetzmann, 62 Hampstead Road, are selling at greatly reduced cost the stock of Messrs. Norman and Stacey. There is a large selection of really solid, useful, and ornamental furniture, which might prove very suitable for the equipment of nurses' homes, domestic offices and rooms, or any institutional purposes. I therefore draw the attention of our readers to the sale, especially as attached to the advantages of purchase is added an offer to store the goods until required, during the year, and to receive payment on the instalment system.

### To Nurses.

We invite contributions from any of our readers, and shall be glad to pay for "Notes on News from the Nursing World," or for articles describing nursing experiences at home or abroad dealing with any nursing question from an original point of view according to length. The minimum payment is 5s. Contributions on topical subjects are specially welcome. Notices of appointments, letters, entertainments, presentations, and deaths are not paid for, but we are always glad to receive them. All rejected manuscripts are returned in due course, and all payments for manuscripts used are made as early as possible after the beginning of each quarter.

### TRAVEL NOTES AND QUERIES.

By our Travel Correspondent.

Accommodation in Turin (A. L. L.).—Thanks for your letter. Am replying by post as our space is precious.

Information About Tyrol (*via* Palestro).—I hope you will see this. Many thanks for kind information which will be most useful.

Norway Travelling (Diana).—The voyage to Norway is sometimes rather rough, but once in the fiords it is quite calm. You would find it enjoyable and not too hot.

Small Places on the Coast Near to Dieppe (C. G. C.).—Please be more explicit, as our space is precious. Tell me exactly how much you can each afford to spend on your holiday and I will then advise. It is rather expensive in that part of France. The Breton coast is cheaper.

Addresses in Belgium (Betty Blue).—I shall be pleased to help you if you will tell me your circumstances, the length of your holiday, and the depth of your purse. I give routes and addresses to suit each individual case, so you must please write me full particulars before I can help you satisfactorily.

Brittany by Dieppe or Calais (A. S. B.).—Reaching Brittany by either these routes seems to me suicidal to the purse. I cannot say the cost exactly, it is not quoted, as no one would dream of adopting either; roughly speaking, I should think it would make £2 10s. or £3 difference, and it would be necessary to sleep on the way. Dinan is a charming place and not dear, you could live there comfortably for about £2 per week, everything included. The passage *via* Southampton and St. Malo in summer is generally good though long. Second return to Dinan £2 5s. 11d.

A Fortnight in Switzerland (Otto).—You do not tell me how much money you have to spend, so I am somewhat in the dark. Glion is rather expensive; you can go near there without choosing so expensive a spot. I should advise a little place I know of up above Lausanne, lovely surroundings, charming scenery (up above the lake), and delightful people. Terms about 5s. to 5s. 6d. a day. If you think this suitable send me a stamped and addressed envelope and I will give you the lady's address. You must use my name. Return fare second class to Lausanne, £4 13s. 4d.

A Holiday at the Lakes (Daga).—You do not give me anything to go upon as to price; the lakes in August are very dear. I like Ambleside as a centre. Write there to Mrs. Dove, Eden Vale, and Mrs. Knipe, Greenend Farm, Hawkshead. I should think it would come to about 35s. per week. If you prefer Windermere write to Mrs. Robinson, Moss Cottage, Brookside, or Mrs. Kirkbride, 4 Milne Terrace, or the least expensive hotel which is the "Queen's." At Ullswater you might get accommodation at the "White Lion" in the village of Patterdale, but it is generally full. There are lodgings to be had but expensive.

Bruges, Antwerp, and Brussels (Old China).—The very cheapest is, as I suggested in this column on the 29th, Tower Bridge to Ostend 6s. Hotel at Bruges, "Panier d'Or" in the market-place. Ghent, Hôtel Aux Armes de Zeelande. Brussels, Hôtel du Rhin, 14 Rue de Brabant. Antwerp, Hôtel du Commerce, 10 Rue de la Bourse. Home by General Steam Navigation Company, second class, about 10s. Could you not overcome the difficulty of spending a night in London by travelling by the night train so as to arrive in London in early morning? Your money will just do the tour as I have planned it, but there will be none to spare. Always ask for rooms on third or fourth floor, and take your lunch out frugally.

A Month's Holiday in July Somewhere Abroad (Tired).—Cheer up, I am sure we can manage something nice for you, but it is a little difficult to make the money stretch over a whole month. However, with management I think it may be done. Should you mind a convent? I know of a very nice one recommended to me by another correspondent with a slender purse. It is quite in the country, and you would have complete rest and peace. It is near to Namur, in Belgium, fairly bracing, I think. Then there is Knokke in Belgium, recommended to "Normandie." St. Malo would, I fear. be beyond you if you remained a month. The only country open to you is Belgium, because of the cheap journey. Tell me what you think of these suggestions and we will arrange further.

Three Weeks in Guernsey (Doreen).—Scotland is quite out of the question; it is far too dear, and I fear you will get nothing in Guernsey for 25s. a week, and as to Torquay it is a pleasant, bright place, but *very dear*. The cheapest place I know of in Guernsey is the Crown Hotel, 5s. 6d. per day. You might go there for a night and look for cheap lodgings. I think very likely the proprietor of the hotel would make an arrangement to board you for 30s. a week if you stayed for some time. You would have ample money for all expenses for three weeks. I should spend half the time in Guernsey and half at Jersey. At the latter place stay at St. Helier's Hotel, "Weigh Bridge." Terms from 5s. 6d. per day. Return ticket to Jersey or Guernsey, second class, £1 17s. 6d.

Three Weeks on the N. French Coast (Normandie).—Anywhere around Dieppe, Calais, or Boulogne would be far beyond your means. I only know of three places on the Continent that could be made to answer your requirements. One is at St. Malo, fare second class return £2 1s. 2d. Board could be had, on the sum you mention, for over two weeks, but not quite for three. This is a seaside place, very bright, gay, and bracing. The second is in Knokke, Belgium, also on the sea, cheaper as to journey but not quite so interesting and agreeable. The third is inland at Bruges. If you like these ideas let me know with a stamped and addressed envelope and I will send you whichever address you choose. I am not permitted to publish them. In either of the Belgian places I think you could stay your full three weeks because the return journey is so cheap.

Holiday in Belgium (A. M. D.).—The money you have is quite sufficient. Do not take circular tickets, it ties you too much and prevents your leaving the beaten track. Take second class single tickets to Antwerp *via* Harwich, cost 15s.; stay three nights in Antwerp. Go to Hôtel du Commerce, 10 Rue de la Bourse. Always ask for rooms on the third or fourth floor. Take your coffee and rolls in the morning and your late dinner at the hotel, but get lunch wherever you may be. Hotel expenses in that way should not come to more than 7 frs. per day, which is 5s. 10d. On leaving Antwerp for Brussels start early, so that you can stop at Malines and see the Cathedral and the Rubens pictures. At Brussels go to Hôtel du Rhin, 14 Rue de Brabant. Stay five days. Much to be seen. Don't waste a day in seeing Waterloo; it is a fraud; so much altered as to be entirely uninteresting. Spend one day in going to Louvain—a very easy excursion—one of the gems of Flanders. From Brussels start early for Ghent, so that you may break your journey at Alost—well worth seeing. At Ghent go to Hôtel d'Allemagne Marché aux Grains. Stay two nights. Leave early, visiting Oudenard and Courtrai, quite easily seen on the way to Ypres, where you may spend the night at Hôtel Châtellenie in the Grande Place. All the interest of Ypres centres in the Grande Place. Be sure to go out after dinner and see it dimly lit up; it is a picture you will never forget. Then go on to Bruges to Hôtel Panier d'Or in the market-place, staying four days. Ostend would be too expensive, and I do not think you would care for it. It is the dullest place in the world unless you go in for Casino gaieties. Boat from Ostend to Dover and rail to London (19s. 10s.). The whole of the railway journeys as I have planned them out will not, I think, exceed £2 second class. If, as I rather think from your letter, you have only 15 days, you must cut off one day at Antwerp and one at Brussels. You will find no difficulty and will enjoy it more than a cut-and-dried circular tour.

Rules in Regard to Correspondence for this Section.—All questioners must use a pseudonym for publication, but the communication must also bear the writer's own name and address as well, which will be regarded as confidential. All such communications to be addressed "Travel Correspondent, 28 Southampton Street, Strand." No charge will be made for inserting and answering questions in the inquiry column, and all will be answered in rotation as space permits. If an answer by letter is required, a stamped and addressed envelope must be enclosed, together with 2s. 6d., which fee will be devoted to the objects of "The Hospital" Convalescent Fund. Ten days must be allowed before an answer can be published.

### Notes and Queries.

**REGULATIONS.**

**The Editor is always willing to answer in this column, without any fee, all reasonable questions, as soon as possible.**

**But the following rules must be carefully observed.**

1. **Every communication must be accompanied by the name and address of the writer.**
2. **The question must always bear upon nursing, directly or indirectly.**

**If an answer is required by letter a fee of half-a-crown must be enclosed with the note containing the inquiry.**

*Registration and Headgear.*

(57) (*a*) Will you please tell me my duty as a certified midwife in connection with "still-birth"? Is it necessary that I should report such cases to the registrar? (*b*) Will you kindly say if there is any uniform covering for the heads of district nurses during the hot, sunny weather in place of the bonnet?—*Nurse D.*

(*a*) Still-born children are not registered but the medical man gives a certificate. (*b*) There is no uniform covering for the heads of district nurses in England, excepting the bonnet.

*Colonial Nursing.*

(58) To whom should I apply for particulars of Colonial nursing? I believe that there is an association which aids nurses desirous of taking up work in the Colonies. Could you invite correspondence from nurses who are, or have been, nursing in the Colonies, as to their experiences?—*L. S. Y.*

Write to the Secretary of the Colonial Nursing Association, Imperial Institute, S.W. If you follow our columns, you will often find articles and letters from nurses in the Colonies.

*Legal Assistance.*

(59) A letter was written to a medical man which contains a libel on a nurse in a responsible position. The opinion of a firm of solicitors, and of counsel, is that an action for damages could be undertaken "with very fair prospect of success." The words used are very strong, and it seems only just that an action should be brought. Can assistance be given in the matter? —*Injured.*

Unless the nurse belongs to an institution we fear that she could not have assistance. But if she is perfectly sure she has been wronged, is determined to clear herself, and can afford to pay the cost, her solicitor being convinced she is legally right, she should not fear the possibility of failure through being unassisted.

*Legal Authority.*

(60) Can any of your correspondents furnish me with the address of the best legal authority on such matters as judicial separation, etc.—*M. B.*

This question is altogether unsuitable for this column, as it does not bear either directly or indirectly upon nursing. See rules. You do not even say if you belong to the nursing profession.

*Private Patient.*

(61) Can you oblige me by letting me know if I should be likely to get a private patient in my private nursing home if I send 10s. 6d. to ———— ? I saw that a home was wanted for an invalid and answered the advertisement. Is this all right, or how do you advise me to get invalids in my country home? I am a nurse, with first-class references from doctors and others.—*M. B.*

Are you sure of the honesty of ———— ? We strongly advise you to be cautious before parting with any money. If your qualifications are good, sooner or later, either by advertising, or privately through doctors and others, you should meet with a private patient.

*X-rays.*

(62) Can you kindly tell me if by paying a fee anybody could go under the *x*-rays in any London hospital? If so, where?—*Nurse G. W.*

A necessitous case could go under the *x*-rays in any of our large London hospitals. If the patient can afford payment the medical attendant will probably suggest some institution or nursing home where the use of the *x*-rays are obtainable.

**Handbooks for Nurses.**

| | Post Free. |
|---|---|
| **"A Handbook for Nurses." (Dr. J. K. Watson.) ...** | **5s. 4d.** |
| **"The Nurses' Dictionary of Medical Terms." ... ...** | **2s. 0d.** |
| **"Massage" (Swedish.) ... ... ... ... ...** | **2s. 6d.** |
| **"Surgical Ward Work and Nursing." ... ... ...** | **3s. 10d.** |
| **"A Complete Handbook of Midwifery." (Watson.) ...** | **6s. 4d.** |

**Of all booksellers or of The Scientific Press, Limited, 28 & 29 Southampton Street, Strand, London, W.C.**

### For Reading to the Sick.

BROTHERHOOD.

O brother man! Fold to thy heart thy brother;
Where pity dwells, the peace of God is there;
To worship rightly is to love each other,
Each smile, a hymn, each kindly deed, a prayer.

Follow with reverent steps the great example
Of Him whose holy work was "doing good";
So shall the wide earth seem our Father's temple,
Each loving life, a psalm of gratitude.

*Whittier.*

"There is no injunction of a general love of men in God's Word: he who is not our brother is our neighbour." The widest Love, in other words, is personal, not a vague undefined sentiment, but the practical recognition of a real claim.

*Bishop Westcott.*

Ought we not to be thoughtful in trying to help on all those special works of thoughtful love which are in the world, such as schools, and penitentiaries, and hospitals, and the like? If God so loved us, if every stage of this creative, redemptive, sanctifying path is marked with thoughtful love, there is, we may be sure, creative, redemptive, sanctifying work for us also to do, whom He condescends to call fellow-workers with Him in the bond and power of love.—*Canon Newbolt.*

Let us reflect that the highest path is pointed out by the pure ideal of those who look up to us, and who, if we tread less loftily, may never look so high again.—*N. Hawthorne.*

Whether any particular day shall bring to you more of happiness or of suffering is largely beyond your power to determine. Whether each day of your life shall give happiness or suffering rests with yourself.—*G. S. Merriam.*

"What is meant by our neighbour we cannot doubt; it is everyone with whom we are brought into contact. First of all, he is literally our neighbour who is next to us in our own family and household; husband to wife, wife to husband, parent to child, brother to sister, master to servant, servant to master. Then it is he who is close to us in our own neighbourhood, in our own town, in our own parish, in our own street. With these all true charity begins. To love and be kind to these is the very beginning of all true religion. But, besides these, as our Lord teaches, it is everyone who is thrown across our path by the changes and chances of life, he or she, whosoever it be, whom we have any means of helping—the unfortunate stranger whom we may meet in travelling, the deserted friend whom no one else cares to look after."—*A. P. Stanley.*

Give me Thine own spirit of self-forgetfulness, that I may be inspired with the power of love. Teach me to lose self-will, that I may be strengthened by a higher will. Let my life be buried in the love of Thee, hid in the sense of Thy presence, absorbed and lost and overshadowed in Thine all-excelling glory.—*Anon.*

